# Eco-friendly fabrication of hydrophobic and breathable nanofibrous membranes *via* molecularly engineered WPU/PAM composites

**DOI:** 10.1039/d5na00467e

**Published:** 2025-08-21

**Authors:** Li Wang, Fajun Peng, Di Jin, Sen Fang, Yan Wang

**Affiliations:** a Hubei College of Chinese Medicine Jinzhou 430043 China 2314452750@qq.com

## Abstract

As demands increase for multifunctional textiles and breathable coatings in high-humidity and high-mobility environments, the development of membranes that combine waterproofing, breathability, and mechanical durability has become a critical challenge. This study presents a novel, organic solvent-free electrospinning approach to fabricate waterborne polyurethane (WPU)-based nanofiber membranes, enhanced by polyacrylamide (PAM) as a dual-functional additive. By leveraging hydrogen bonding interactions between the –COO^−^, –NHCOO– groups in WPU and the –CONH_2_ groups in PAM, the resulting composite achieved stable electrospinning, improved fiber morphology, and a significantly higher water contact angle (86.9°), compared to conventional WPU/PVA systems (<10°). The optimized WPU/PAM-3 membrane maintained high air permeability and excellent tensile strength, while also showing low water absorption (∼18%) and strong structural stability under thermal, mechanical, and environmental cycling. Structural analyses *via* FTIR, XRD, and XPS confirmed enhanced interfacial compatibility and molecular interaction. Notably, this work eliminates the need for volatile organic solvents and hygroscopic additives like PVA, solving common limitations in traditional WPU systems. The resulting membrane offers a sustainable, high-performance solution for protective textiles, medical materials, and flexible barrier coatings, marking a significant advancement in eco-friendly, breathable membrane technology.

## Abstract 2

With the increasing demand for versatile breathable materials, developing membranes that are both waterproof, breathable, and durable has become a key challenge in addressing high-humidity, high-activity environments.

## Graphical abstract

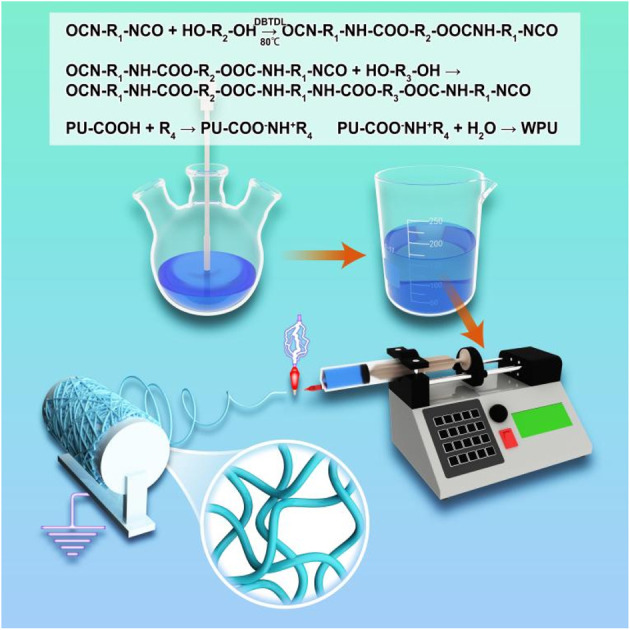

## 1. Introduction

The escalating demand for advanced protective textiles, driven by global industrialization and increased health awareness, has necessitated the development of materials that balance seemingly contradictory requirements: impermeability to liquid water, efficient moisture vapor transmission, and mechanical robustness.^1–6^ While conventional solvent-based polyurethanes have been widely employed in waterproof membranes, their utility is fundamentally constrained by two interrelated challenges. First, the tortuous pore structures inherent to cast polyurethane films severely limit water vapor permeability, leading to thermal discomfort during prolonged wear.^7–9^ More critically, their synthesis and processing predominantly rely on volatile organic compounds (VOCs) such as dimethylformamide (DMF) and toluene, which account for over 60% of the carbon footprint in polyurethane manufacturing and pose significant neurotoxic risks to workers.^10–13^ These dual deficiencies in performance sustainability and environmental compatibility have propelled WPU systems to the forefront of research, as their aqueous dispersion eliminates VOC emissions and aligns with circular economy principles.^14,15^

Electrospinning technology offers a promising strategy to enhance the breathability of WPU membranes by creating nanofibrous networks with tunable porosity. However, the inherent hydrophilicity of typical WPU formulations, characterized by water contact angles less than 60°, often necessitates blending with hygroscopic polymers, such as PVA, to achieve sufficient spinnability.^16–20^ This approach, however, introduces environmental challenges. PVA's high solubility leads to fiber disintegration when exposed to humidity, and its non-biodegradability raises significant concerns regarding waste disposal at the end of its lifecycle. Additionally, the water contact angle of electrospun fibers composed of WPU/PVA blends is often below 10°, which limits their moisture resistance.^21–25^ Recent advances in polymer blending have suggested that replacing PVA with amphiphilic copolymers can help modulate both solution rheology and surface energetics. However, the influence of intermolecular interactions between the urethane groups of WPU and blending polymers on fiber formation dynamics and wet stability remains insufficiently explored for WPU-based systems.^26–29^ Wang *et al.*^30^ utilized PCL-*b*-WPU block copolymers in electrospinning, where the membranes exhibited a water contact angle that rapidly decreased to 0° within 120 seconds, indicating high wettability but limited water resistance and stability. Similarly, Alonso-Lerma *et al.*^31^ reinforced WPU with cellulose nanocrystals (CNCs) to improve thermomechanical properties, resulting in a water contact angle of approximately 55°. While this modification enhanced the material's stability, it still struggled to achieve higher water resistance, limiting its practical applications in moisture-proof textiles. In this study, we present a sustainable manufacturing paradigm for high-performance WPU membranes *via* molecular engineering and organic solvent-free electrospinning. A carboxylated anionic WPU dispersion was synthesized through a three-step process. The prepolymer was formed by reacting (IPDI) with poly(butylene adipate) (PBA). Dimethylolpropionic acid (DMPA) was then incorporated as a hydrophilic chain extender to introduce carboxylic acid groups (–COOH) into the polymer backbone. 1,4-Butanediol (BDO) was subsequently added as a chain extender to facilitate further polymer chain growth. Triethylamine (TEA) was used as a neutralizing agent to protonate approximately 95% of the pendant –COOH groups, resulting in the formation of carboxylate anions (–COO^−^), which facilitated the stable aqueous dispersion (solid content: 30 wt%). Innovatively, PAM was introduced as a multifunctional co-blending component, fulfilling dual roles: (i) as a viscoelasticity modifier, where it interacts with the –COO^−^ of WPU, thereby enhancing the solution's rheological properties and ensuring stable electrospinning without the need for organic additives, and (ii) as a hydrophobicity regulator, where hydrogen bonding between PAM's amide (–CONH_2_) groups and WPU carbamate (–NHCOO–) groups improves the moisture resistance of the fibers. This interaction results in fibers with enhanced stability under humid conditions and controlled hydrophobicity, achieving superior water contact angles. The synergistic effect of these modifications leads to membranes with exceptional hydrostatic pressure and high water vapor transmission rates, outperforming conventional WPU/PVA membranes in both aspects.

## 2. Experiments

### 2.1 Materials

Experimental reagents and their sources are listed in Table 1.

**Table 1:** Chemical reagents

Reagent name	Specifications	Source
IPDI	99%	Aladdin Reagents (Shanghai) Co., Ltd
PBA	99%	Aladdin Reagents (Shanghai) Co., Ltd
DMPA	99%	Aladdin Reagents (Shanghai) Co., Ltd
BDO	99%	Aladdin Reagents (Shanghai) Co., Ltd
TEA	99%	Aladdin Reagents (Shanghai) Co., Ltd
PAM	Molecular weight ∼12 000 000	Aladdin Reagents (Shanghai) Co., Ltd
DMF	AR	Aladdin Reagents (Shanghai) Co., Ltd
DBTDL	95%	Aladdin Reagents (Shanghai) Co., Ltd

### 2.2 Synthesis of WPU

In a three-neck round-bottom flask, 18 g of PBA was placed and vacuum-dried at 120 °C for 2 hours to remove any moisture. Afterward, the temperature was reduced to 60 °C, and 9.6 g of IPDI was added. Additionally, 0.8% of the total material mass of DBTDL was added as a catalyst. The mixture was then heated to 80 °C to pre-polymerize for 3 hours. Following the pre-polymerization, 1.48 g of DMPA was introduced, and the reaction continued at 80 °C for 1 hour to introduce hydrophilic groups into the molecular chain. Subsequently, 0.72 g of BDO was added as a chain extender, and the reaction was carried out at 80 °C for 30 minutes to promote chain growth. After the reaction, the temperature was lowered to 50 °C, and 0.96 g of TEA was added to neutralize the resin and form a salt. The reaction was continued for 30 minutes, and deionized water was added to emulsify the resin, yielding a WPU emulsion with a solid content of 30%.

### 2.3 Electrospinning of WPU

The WPU emulsion with a solid content of 30 wt% was first prepared for electrospinning. The emulsion was transferred into a syringe equipped with a metal needle, and the electrospinning process was performed under controlled conditions. The syringe was connected to a high-voltage power supply, and the electrospinning setup was operated with the following parameters: a voltage of 18–22 kV, a feeding rate of 0.2 mL h^−1^, and a needle-to-collector distance of 15 cm. The collector was grounded, and the electrospun fibers were collected on a rotating drum to ensure uniformity in fiber alignment. The temperature and humidity of the electrospinning environment were maintained at 25 °C and 30%, respectively, to prevent any environmental influence on the fiber morphology.

After the electrospinning process was completed, the obtained nanofibrous membrane was further dried at 50 °C for 12 hours to remove any residual water and to improve the mechanical properties of the membrane. The resulting membrane was then stored in a desiccator to maintain its structural integrity until further characterization. The electrospun membranes were labeled according to the mass percentage of the additive polymer relative to the mass of WPU. Specifically, “WPU/PAM-3” denotes a membrane in which PAM accounts for 3 wt% of the WPU mass, while “WPU/PAM-6” represents a 6 wt% PAM loading. For comparison, membranes prepared using polyvinyl alcohol were labeled as “WPU/PVA-3” and “WPU/PVA-6”, corresponding to 3 wt% and 6 wt% of PVA relative to the WPU content, respectively.

### 2.4 Characterization

The chemical structure of the synthesized WPU and its composite membranes was analyzed using Fourier transform infrared spectroscopy (FTIR, Nicolet iS50, Thermo Fisher Scientific) in the range of 4000–500 cm^−1^. Crystallinity and molecular ordering were evaluated by X-ray diffraction (XRD, Bruker D8 Advance) with Cu Kα radiation (*λ* = 1.5418 Å), scanned over a 2*θ* range of 5°–70°. Surface elemental composition and chemical states were further examined using X-ray photoelectron spectroscopy (XPS, ESCALAB 250Xi, Thermo Fisher), with high-resolution scans of C 1s and O 1s regions. The morphological characteristics of electrospun membranes were observed *via* scanning electron microscopy (SEM, JEOL JSM-IT500), and fiber diameter distribution was calculated using ImageJ software based on SEM images. Porosity and pore size distribution were analyzed by capillary flow porometry (Porous Materials Inc.). Air permeability was measured using a Gurley-type apparatus under standardized conditions. Static water contact angles were measured using a contact angle goniometer (Krüss DSA100) to evaluate surface wettability. Water absorption tests were performed by immersing membrane samples in deionized water for 24 hours and recording the weight change. Thermogravimetric analysis (TGA, NETZSCH STA449F3) was conducted under a nitrogen atmosphere from 30 °C to 600 °C at a heating rate of 10 °C min^−1^ to assess thermal stability. Mechanical properties were tested using a universal tensile testing machine (Instron 3343) at a crosshead speed of 10 mm min^−1^. To assess environmental reliability, membranes were subjected to thermal holding, thermal cycling (25–70 °C, 200 cycles), tensile cycling (up to 100% strain, 50 cycles), and natural aging for 28 days under ambient conditions, with air permeability recorded as the primary performance metric throughout the aging process. Cell viability was evaluated using a CCK-8 assay to test the L929 cell viability after co-culturing with the membranes for 24 hours. Fluorescent images of live and dead cells were captured using a laser confocal microscope to further assess the biological compatibility of the membranes. The biocompatibility tests were conducted on the Hangzhou Yanqu Information Technology Co., Ltd platform. The L929 cell line sample was purchased from Wuhan Pricella Biotechnology Co., Ltd, and its ORID number is CVCL_0462. Additionally, UV aging tests were conducted using a UV340 ultraviolet lamp to expose the samples to UV radiation for a total of 120 hours to assess the durability and stability of the membranes under UV exposure.

## 3. Results and discussion

### 3.1 Design of environmentally friendly WPU nanofibers for enhanced waterproof and moisture-permeable properties

**Fig. 1:**
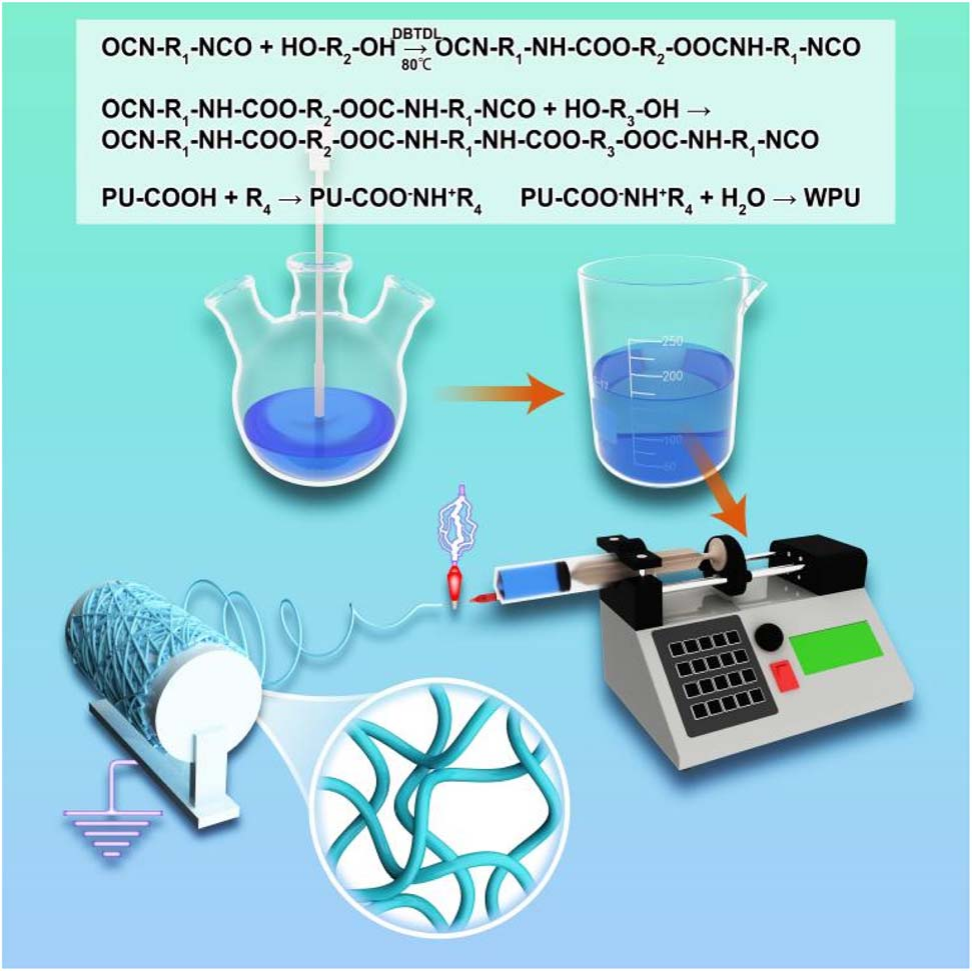
Schematic of the synthesis and electrospinning process of WPU nanofibers.

This study developed a nanofiber membrane based on carboxylated anionic WPU blended with PAM through molecular design and organic solvent-free electrospinning technology, aiming to synergistically enhance both waterproof and breathable properties. The main chain of WPU was functionalized by introducing –COOH and –NHCOO– groups, imparting amphiphilicity to the material. PAM's –CONH_2_ groups formed dynamic crosslinked networks with the –COO^−^ and carbamate (–NHCOO–) groups of WPU through hydrogen bonding. These intermolecular interactions not only enhanced the rheological properties of the solution, ensuring the stability of the jet during organic solvent-free electrospinning, but also significantly improved the hydrophobicity of the fiber surface *via* microphase separation, increasing the water contact angle to over 85°, far exceeding that of traditional WPU/PVA systems (<10°). Additionally, the incorporation of PAM enhanced the membrane's stability in humid environments, addressing the performance degradation of traditional WPU membranes due to excessive hydrophilicity in moist conditions. By optimizing the blending ratio of WPU and PAM, we successfully fabricated nanofiber membranes with a high water contact angle, while maintaining excellent moisture permeability and mechanical strength. These improved properties allow the waterborne polyurethane membrane to achieve a balanced performance between waterproofing and breathability, offering broad application prospects. Particularly, this material demonstrates superior overall performance compared to traditional WPU/PVA membranes, making it suitable for protective textiles, medical materials, and other fields that require both waterproofing and breathability. The uniqueness of this nanofiber membrane arises from the optimization of its microstructure and the innovation in molecular design. Through organic solvent-free electrospinning and the blending of WPU with PAM, this membrane not only resolves the performance decline of traditional polyurethane membranes in humid environments but also enhances both its environmental friendliness and functionality. Experimental results indicate that the waterborne polyurethane membrane outperforms traditional WPU and PVA systems in terms of waterproofing, moisture permeability, and mechanical strength, demonstrating significant application potential. The specific chemical reaction equations and experimental procedure are shown in Fig. 1.

### 3.2 Morphology of electrospun WPU nanofibers

**Fig. 2:**
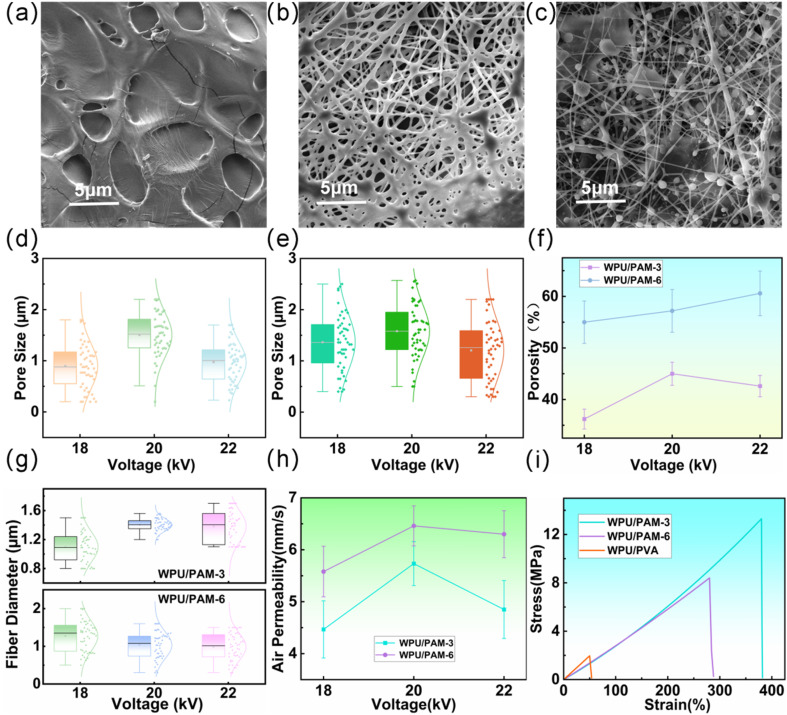
Morphology and properties of electrospun WPU/PAM-3 nanofibers under different voltages. (a) SEM image of WPU/PAM-3 nanofibers at 18 kV, showing thicker fibers with less defined pore structure. (b) SEM image of WPU/PAM-3 nanofibers at 20 kV, showing finer, more uniform fibers and a more organized fibrous network. (c) SEM image of WPU/PAM-3 nanofibers at 22 kV. (d) Pore size distribution of WPU/PAM-3 at different voltages. (e) Pore size distribution of WPU/PAM-6 at different voltages. (f) Comparison of porosity for WPU/PAM-3 and WPU/PAM-6 under different voltages. (g) Fiber diameter distribution of WPU/PAM-3 and WPU/PAM-6 at different voltages. (h) Air permeability test results for WPU/PAM-3 and WPU/PAM-6. (i) Stress–strain curves of WPU/PAM-3, WPU/PAM-6, and traditional WPU/PVA electrospun fibers.

The morphology of electrospun WPU/PAM nanofibers was examined under different voltages (18 kV, 20 kV, and 22 kV) using SEM, with the resulting fiber structures shown in Fig. 2a–c.

At 18 kV (Fig. 2a), the fibers did not fully form, and the fiber diameter was relatively large, without a well-defined pore structure. As the voltage was increased to 20 kV (Fig. 2b), the fibers became finer, with more uniform and well-organized fibrous networks forming. However, when the voltage was raised to 22 kV, the increased electric field strength resulted in excessive force on the electrospinning solution, leading to instability. Although finer fibers were produced, a bead-on-string phenomenon also appeared due to the excessive stretching forces at this voltage.

Fig. 2d and e present the pore size distribution of WPU/PAM-3 and WPU/PAM-6 at different voltages. With increasing voltage, the pore size of both WPU/PAM-3 and WPU/PAM-6 exhibited a trend of initially increasing and then decreasing, with WPU/PAM-6 samples having significantly larger pores than WPU/PAM-3. This is consistent with the SEM images, where the pore size observed in the fibers correlates with the applied voltage and PAM concentration. Fig. 2f compares the porosity of WPU/PAM-3 and WPU/PAM-6 at various voltages. Higher porosity indicates better breathability and moisture vapor transmission capability of the membranes. In the case of WPU/PAM-3, the fibers were thicker, leading to lower porosity compared to WPU/PAM-6. Specifically, the porosity of WPU/PAM-3 was 45% at 20 kV, while WPU/PAM-6 showed a porosity of 61% at 22 kV, indicating improved air permeability for the latter.

The variation in fiber diameter (Fig. 2g) reveals that WPU/PAM-6 fibers were finer at 20 kV and 22 kV compared to WPU/PAM-3. This improvement is attributed to the higher PAM content, which enhances the stability of the electrospinning process. The effect of voltage on fiber diameter is also evident, with thicker fibers being produced at 18 kV and finer fibers at higher voltages (20 kV and 22 kV), which is typically due to enhanced elongation under stronger electric fields. Fig. 2h presents the air permeability test data, indicating that WPU/PAM-6 samples exhibited significantly higher air permeability than WPU/PAM-3 at all voltages, suggesting superior breathability. This improvement is attributed to the higher porosity and smaller fiber diameter in WPU/PAM-6, which contribute to better air transmission through the membrane.

Finally, mechanical performance was evaluated using stress–strain curves (Fig. 2i). Both WPU/PAM-3 and WPU/PAM-6 samples showed superior tensile strength and elongation at break compared to traditional WPU/PVA electrospun fibers. Particularly, WPU/PAM-3 exhibited the best tensile strength and elongation at break. In contrast, traditional WPU/PVA electrospun fibers demonstrated poor tensile performance, primarily because PVA, with its strong hydrophilicity, forms a rigid polymer network when blended with WPU. During stretching, the rigidity of the PVA network fails to effectively distribute the applied stress, leading to brittle fracture. Moreover, the strong hydrogen bonding between PVA molecules reduces its extensibility, making it less flexible and less capable of elongation compared to PAM. Consequently, the inclusion of PVA significantly diminishes the membrane's stretching ability, limiting its flexibility and toughness. In contrast, the blend of PAM with WPU resulted in a more flexible composite network, thanks to the inherent flexibility of PAM molecules and their interaction with WPU chains. This network effectively disperses the stress during stretching, enhancing the film's ductility and toughness, thereby maintaining higher tensile strength and elongation at break. The addition of PAM allows the membrane to better absorb and distribute external stress, preventing brittle fracture and improving the overall mechanical performance.

In conclusion, electrospun WPU/PAM nanofibers exhibit tunable morphological characteristics, including fiber diameter, pore size, porosity, and mechanical performance. These properties are significantly influenced by electrospinning voltage and PAM concentration. Although WPU/PAM-6 samples outperform WPU/PAM-3 in terms of porosity and air permeability, their fiber network structure is not ideal, and their tensile properties are inferior to those of WPU/PAM-3. Therefore, considering these factors, WPU/PAM-3 will be selected for subsequent experiments, as it offers a balanced performance for membrane materials.

### 3.3 Materials characterization and structural analysis

**Fig. 3:**
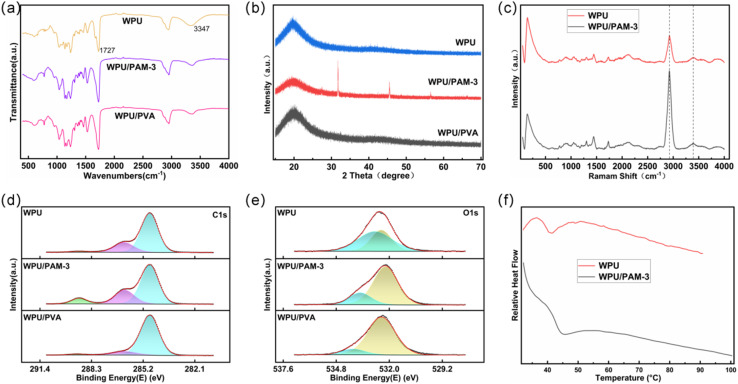
(a) FTIR spectra of WPU, WPU/PAM-3, and WPU/PVA nanofiber membranes. (b) XRD patterns of WPU, WPU/PAM-3, and WPU/PVA membranes. (c) Raman spectra of WPU and WPU/PAM-3 nanofibers. (d) High-resolution C 1s XPS spectra of WPU, WPU/PAM-3, and WPU/PVA. (e) High-resolution O 1s XPS spectra of WPU, WPU/PAM-3, and WPU/PVA. (f) DSC curves of WPU and WPU/PAM-3 nanofibers.

The structural evolution and elemental composition of the WPU-based nanofiber membranes were systematically investigated by FTIR, XRD, and XPS, as shown in Fig. 3. In the FTIR spectra, the pure WPU sample exhibits characteristic absorption bands at 1727 cm^−1^ and 3347 cm^−1^, corresponding to the C=O stretching and N–H stretching vibrations of the carbamate groups, respectively, confirming the successful incorporation of urethane functionalities. Notably, no characteristic –NCO stretching vibration band appears near 2270 cm^−1^, indicating that the residual isocyanate groups were completely reacted during the synthesis process, thereby ensuring high chemical purity. Upon incorporation of PAM, the WPU/PAM-3 sample shows a slight shift and broadening of the N–H stretching band, suggesting the formation of hydrogen bonds between the amide groups (–CONH_2_) of PAM and the carbamate groups (–NHCOO–) of WPU. In contrast, the WPU/PVA sample exhibits sharper and less broadened peaks, reflecting weaker interfacial interactions and inferior molecular compatibility compared to the WPU/PAM-3 system. The XRD patterns shown in Fig. 3b provide further insights into the structural organization of the membranes. Pure WPU displays a broad amorphous diffraction peak centered around 2*θ* ≈ 20°. The incorporation of PAM results in a slight sharpening and intensity enhancement of this peak in the WPU/PAM-3 sample, indicating improved microphase separation or short-range molecular ordering facilitated by hydrogen bonding. Conversely, the WPU/PAM sample exhibits additional sharp peaks, characteristic of the intrinsic semi-crystalline structure of PAM,^32,33^ suggesting lower homogeneity in blending and more pronounced phase segregation. The Raman spectra of WPU and WPU/PAM-3 samples show significant differences (Fig. 3c), particularly at 2950 cm^−1^ and 3400 cm^−1^. The CH peak at 2950 cm^−1^ becomes sharper, and the NH peak at 3400 cm^−1^ also exhibits increased intensity and sharpness in the WPU/PAM-3 composite. These changes are attributed to the hydrogen bonding interactions between the –COO^−^ groups of WPU and the –CONH_2_ groups of PAM. The formation of these hydrogen bonds enhances the molecular interactions within the composite, leading to a more defined peak structure in the Raman spectra. The XPS analysis, shown in Fig. 3d and e, complements the FTIR and XRD results by elucidating the surface chemical environments. In the C 1s spectra, all samples display peaks at binding energies of approximately 284.8 eV (C–C/C–H), 286.3 eV (C–O/C–N), and 288.6 eV (O–C=O). In the WPU/PAM-3 sample, a significant increase in the relative intensities of the C–N and O–C=O signals confirms the successful incorporation of amide and carboxylate functionalities from PAM. Furthermore, the O 1s spectra reveal two major peaks at around 531.6 eV and 533.2 eV, corresponding to C=O and C–O–H/C–O–C bonds, respectively. The increased proportion of C=O bonds in the WPU/PAM-3 sample further supports the formation of stable hydrogen bonds between PAM and WPU chains. From the DSC data shown in Fig. 3f, the glass transition temperature of the synthesized WPU is 40 °C, while the *T*_g_ of the WPU/PAM-3 sample increases to 45 °C. This shift is attributed to the formation of hydrogen bonds between the –COO^−^ groups of WPU and the –CONH_2_ groups of PAM, which enhances the intermolecular interactions. The hydrogen bonding increases the rigidity of the polymer chains, restricting their mobility and thus raising the glass transition temperature. This result suggests that the incorporation of PAM leads to a more ordered and stable molecular structure within the composite, improving its thermal properties. These comprehensive analyses verify that the blending of PAM effectively alters the chemical environment of WPU, enhances molecular interactions, and contributes to the structural stability and improved performance of the nanofiber membranes.

### 3.4 Wettability and environmental reliability

**Fig. 4:**
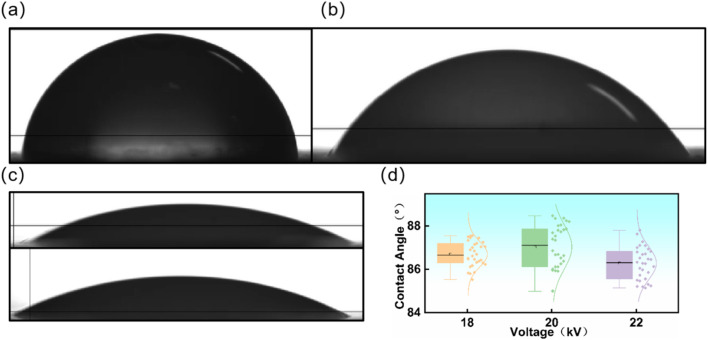
(a) Static water contact angle of WPU/PAM-3 membrane. (b) Water contact angle of WPU/PAM-6 membrane. (c) Water contact angles of WPU/PVA-3 and WPU/PVA-6 membranes; (d) statistical distribution of water contact angles for WPU/PAM-3 membranes electrospun under 18 kV, 20 kV, and 22 kV, respectively.

The static water contact angle results of the electrospun membranes are presented in Fig. 4. As shown in Fig. 4a, the WPU/PAM-3 membrane exhibited a contact angle of 86.9°, while the WPU/PAM-6 sample in Fig. 4b showed a slightly lower value of 61.7°. Despite the higher PAM content in WPU/PAM-6, its increased hydrophilicity is attributed to the intrinsic water affinity of PAM, which is widely used in hydrogel formation due to its abundant –CONH_2_ groups that readily form hydrogen bonds with water molecules. When incorporated at an appropriate concentration, PAM can effectively interact with the –COO^−^ and –NHCOO– of WPU to enhance solution viscosity and spinnability. However, excessive PAM loading introduces additional hydrophilic domains, counteracting the hydrophobic enhancement and thereby reducing the overall contact angle.

As illustrated in Fig. 4d, the water contact angles of WPU/PAM-3 membranes electrospun under different voltages exhibited only minor variations, with values fluctuating between 84.5° and 88.2°. The minimal impact of voltage on wettability can be ascribed to the fact that all samples possessed a uniform fiber surface chemistry, as they were prepared from identical formulations. Furthermore, the voltage-induced differences in fiber morphology and porosity were not sufficient to significantly alter the macroscopic wetting behavior. Thus, within the studied voltage range, the electrospinning parameters had negligible influence on the surface hydrophobicity of the resulting membranes.

**Fig. 5:**
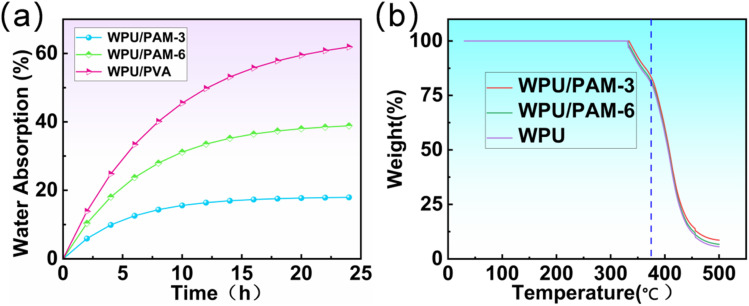
(a) Water absorption profiles of WPU/PAM-3, WPU/PAM-6, and WPU/PVA membranes over 24 hours of immersion. (b) Thermogravimetric analysis curves of WPU/PAM-3, WPU/PAM-6, and pure WPU samples.

The water absorption behavior of the electrospun membranes was investigated by immersing the samples in water for varying durations, as shown in Fig. 5a. The results reveal that WPU/PAM-3 exhibited the lowest water absorption rate among the samples, maintaining a relatively stable absorption curve, with a final water uptake of approximately 18%. In comparison, WPU/PAM-6 showed a higher absorption, reaching around 39%, which can be attributed to the increased hydrophilic character of PAM at higher concentrations. On the other hand, WPU/PVA, with its highly hydrophilic nature, demonstrated the highest water absorption, exceeding 60% after 24 hours of immersion. These findings highlight the critical role of PAM content in regulating the water absorption capacity of the membranes, where a moderate amount of PAM can maintain reasonable hydrophobicity and minimize excessive water uptake. TGA of the WPU/PAM-3 sample, shown in Fig. 5b, reveals key insights into the thermal stability of the membrane. The first significant weight loss, starting around 320 °C, corresponds to the degradation of WPU's soft segment and PAM. At approximately 380 °C, the degradation rate sharply increases as the hard segment of WPU begins to decompose. This two-step degradation process indicates that WPU/PAM-3 maintains reasonable thermal stability up to this point, with a rapid loss of weight occurring only at higher temperatures. Notably, the thermal decomposition behaviors of WPU/PAM-3 and WPU/PAM-6 are virtually identical, so only the WPU/PAM-3 thermogram is presented for clarity. These results confirm that the addition of PAM does not significantly alter the thermal stability of the WPU matrix.

**Fig. 6:**
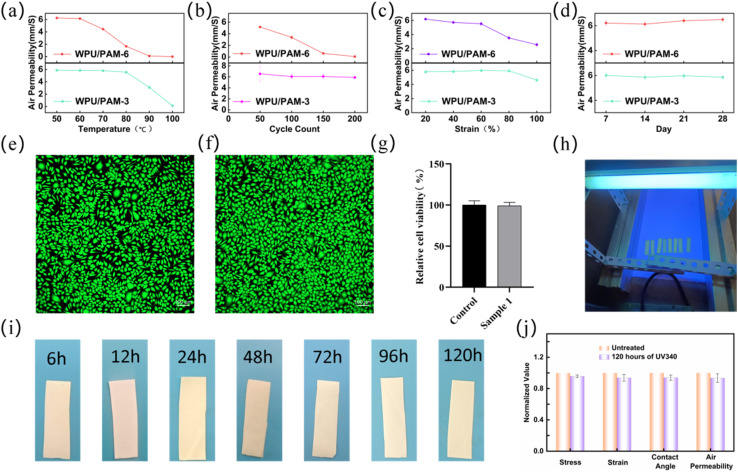
(a) Air permeability of WPU/PAM-3 and WPU/PAM-6 membranes after being held at designated temperatures for 12 hours. (b) Thermal cycling test results (room temperature to 70 °C), showing air permeability changes over 200 cycles. (c) Air permeability after 50 cycles of tensile stretching at different strain levels. (d) Natural aging performance monitored over 28 days under ambient conditions. (e) Fluorescent images of cell viability and death staining, where the left panel (e) represents the control group and the right panel (f) shows the co-culture sample group. (g) CCK-8 assay results illustrating the cell viability of the two groups. (h) Macroscopic image showing the ongoing UV aging test. (i) Macroscopic images of the best-performing sample after 120 hours of UV exposure. (j) Normalized data for stress–strain behavior, water contact angle, and air permeability of the UV-aged samples compared with untreated samples.

To evaluate the environmental and mechanical durability of the electrospun membranes, WPU/PAM-3 and WPU/PAM-6 samples were subjected to a series of aging tests, including thermal holding, thermal cycling, tensile cycling, and natural aging, with air permeability used as the primary performance indicator. As shown in Fig. 6a, samples were placed at fixed temperatures for 12 hours before measurement. WPU/PAM-3 exhibited stable air permeability up to 80 °C, with noticeable decline only emerging beyond this threshold. In contrast, WPU/PAM-6 showed a marked reduction in air permeability starting as early as 60 °C, indicating that higher PAM content compromises thermal stability, likely due to the greater presence of thermally labile hydrophilic segments. Based on these observations, thermal cycling tests were conducted between room temperature and 60 °C to simulate practical heating fluctuations (Fig. 6b). WPU/PAM-3 maintained nearly constant permeability across 200 cycles, suggesting strong structural resilience to repeated thermal stress. Conversely, WPU/PAM-6 displayed a progressive decline in permeability with increasing cycles, reflecting cumulative damage and internal fiber disruption. Mechanical cycling durability was further evaluated by subjecting membranes to 50 cycles of tensile deformation under various strain levels (Fig. 6c). WPU/PAM-3 retained over 80% of its initial air permeability even at 100% strain, highlighting its excellent mechanical robustness. By contrast, WPU/PAM-6 experienced a substantial drop in performance as strain increased, likely due to structural fatigue and reduced fiber cohesion under repeated extension. Lastly, natural aging stability was monitored over 28 days at room temperature and ambient humidity (Fig. 6d). Both WPU/PAM-3 and WPU/PAM-6 exhibited minimal changes in air permeability, confirming their chemical and physical stability under standard environmental exposure. In conclusion, WPU/PAM-3 demonstrated superior durability across all conditions tested, reinforcing its suitability as a robust, breathable membrane material for long-term applications requiring thermal, mechanical, and environmental stability.

The biological compatibility of materials is crucial for their potential applications in medical and biotechnological fields, where safe interaction with living cells is paramount. As shown in Fig. 6e and f, after 24 hours of co-culturing with cells, no significant cell death was observed, indicating the materials' favorable biocompatibility. This observation was further confirmed by the CCK-8 assay (Fig. 6g), which demonstrated high cell viability, supporting the low cytotoxicity of the WPU/PAM membranes. In addition to their biological performance, the UV durability of the membranes was also assessed. Macroscopic images were captured during the ongoing UV aging process for 120 hours (Fig. 6h), providing insights into the membranes' stability under UV exposure. Following 120 hours of UV exposure, the sample showed no significant changes, indicating excellent UV stability (Fig. 6i). This observation was further supported by the performance tests shown in Fig. 6j, where the performance of the UV-exposed sample exhibited less than a 10% variation compared to the original, demonstrating the material's strong stability against UV-induced degradation.

## 4. Conclusion

This study successfully established a organic solvent-free electrospinning strategy to fabricate high-performance nanofibrous membranes based on carboxylated WPU and PAM. Through molecular engineering and hydrogen bonding interactions between –COO^−^, –NHCOO–, and –CONH_2_ groups, the resulting WPU/PAM-3 membranes exhibited significant improvements in hydrophobicity, moisture permeability, and mechanical resilience. Specifically, the WPU/PAM-3 composite achieved a water contact angle of 86.9°, markedly higher than that of conventional WPU/PVA systems (<10°), while maintaining excellent air permeability and tensile properties. These results highlight the effectiveness of amphiphilic structure design and fiber morphology optimization in achieving membranes with a well-balanced combination of waterproofness and breathability, suitable for use in dynamic and humid environments.

Importantly, this work overcomes the common limitations of waterborne polyurethane membranes, such as excessive hydrophilicity and poor wet-state performance, by introducing PAM as a dual-functional additive that modulates solution rheology and fiber surface properties without relying on volatile organic solvents. However, an excessive PAM concentration led to decreased hydrophobicity and mechanical uniformity due to increased water affinity, indicating the necessity of optimizing the blending ratio for practical applications.

Future research should investigate the long-term performance of these membranes in real-world environments, including exposure to fluctuating humidity and temperature conditions. Additionally, scaling the organic solvent-free electrospinning process and exploring multi-functional enhancements, such as antimicrobial or flame-retardant capabilities, could broaden the applicability of WPU/PAM membranes in advanced protective textiles, medical materials, and breathable coatings.

## Conflicts of interest

There are no conflicts to declare.

## Data Availability

The data that support the findings of this study are available from the corresponding author upon reasonable request.
